# Regulated necrosis in COVID-19: A double-edged sword

**DOI:** 10.3389/fimmu.2022.917141

**Published:** 2022-08-25

**Authors:** Chen Sun, Yunze Han, Ruoyu Zhang, Simon Liu, Jing Wang, Yuqing Zhang, Xuemei Chen, Chao Jiang, Junmin Wang, Xiaochong Fan, Jian Wang

**Affiliations:** ^1^ Department of Pain Medicine, The First Affiliated Hospital of Zhengzhou University, Zhengzhou, China; ^2^ Medical Genomics Unit, National Human Genome Research Institute, Bethesda, MD, United States; ^3^ Department of Human Anatomy, School of Basic Medical Sciences, Zhengzhou University, Zhengzhou, China; ^4^ Department of Neurology, Fifth Affiliated Hospital of Zhengzhou University, Zhengzhou, China

**Keywords:** COVID-19, necrosis, mechanisms, biomarkers, clinical treatment

## Abstract

COVID-19 caused by SARS-CoV-2 can cause various systemic diseases such as acute pneumonia with cytokine storm. Constituted of necroptosis, pyroptosis, and ferroptosis, regulated necrosis constitutes the cell death patterns under the low apoptosis condition commonly observed in COVID-19. Regulated necrosis is involved in the release of cytokines like TNF-α, IL-1 β, and IL-6 and cell contents such as alarmins, PAMPs, and DAMPs, leading to more severe inflammation. Uncontrolled regulated necrosis may explain the poor prognosis and cytokine storm observed in COVID-19. In this review, the pathophysiology and mechanism of regulated necrosis with the double-edged sword effect in COVID-19 are thoroughly discussed in detail. Furthermore, this review also focuses on the biomarkers and potential therapeutic targets of the regulated necrosis pathway in COVID-19, providing practical guidance to judge the severity, prognosis, and clinical treatment of COVID-19 and guiding the development of clinical anti-SARS-CoV-2 drugs.

## Highlights

1. Regulated necrosis plays a critical role in the pathologic process of COVID-19.

2. The mechanisms and “double-edged sword” effects of regulated necrosis are discussed.

3. The potential therapeutic targets can guide the development of anti-SARS-CoV-2 drugs.

## 1 Introduction

An unprecedented worldwide spread of coronavirus-2 of the severe acute respiratory syndrome (SARS-CoV-2), the cause of coronavirus disease 2019 (COVID-19), has imposed tough challenges on the health and medical infrastructure around the world (1). As of 1 May 2022, 510 million people had been diagnosed with COVID-19, including 6 million deaths worldwide (2). The initial clinical manifestations are mainly nonspecific respiratory syndromes (3–5) followed by complex complications, including multiple organ failure, septicemia, and cytokine storm (6–8). SARS-CoV-2 infects the host with the cell receptor angiotensin-converting enzyme-2 (ACE2) through respiratory droplets and causes various pathophysiological changes and syndromes. Cell metabolism is interpreted as the trigger for cell death through multiple pathways that coexist in COVID-19 (9). More components and increased cell damage lead to systemic inflammatory response syndrome (SIRS) and a SIRS-like immune response (10). With the aggravation of the now dysfunctional inflammatory system, inflammatory monocytes and neutrophils increase, and lymphocytes decrease markedly (6, 11, 12). Cytokine storms and a high burden of systemic inflammation accelerate subclinical disorders and lead to complications through inflammatory cell death (12). In this process, regulated necrosis plays a vital role in the pathophysiology of COVID-19 (13); exploring its connection to COVID-19 can provide a crucial theoretical basis for our treatment of COVID-19.

Necrosis is an old concept that has recently gained new attention (14). Traditional necrosis, characterized by organelle disintegration, oncosis, degeneration of proteins and enzymes *in vivo*, and plasma membrane rupture (15), evokes inflammatory responses by releasing damage-associated molecular patterns (DAMP) that trigger an immune response known as necroinflammation (16). During inflammation, necrotic cells and neutrophils exude lysosomal enzymes, promoting further necrosis and local parenchymal lysis of multiple cells simultaneously (17).

More recently, regulated necrosis has emerged as a revolutionary concept (18). Unlike traditional necrosis, which is understood to be a disordered, passive, gene-independent, and pathological process, regulated necrosis is genetically programmed. It biochemically represents various signaling pathways, such as kinase-mediated necroptosis, gasdermin-mediated necrosis downstream of inflammasomes, and an iron-catalyzed mechanism (14, 19, 20). Regulated necrosis comes in different forms, such as necroptosis, pyroptosis, and ferroptosis, all of which play an essential role in host defense and maintaining tissue homeostasis (18). Furthermore, regulated necrosis also contributes to the pathophysiology of various inflammatory, infectious, tumor, and degenerative diseases (21, 22). Like apoptosis, regulated necrosis can be controlled by specific molecular modulations on therapeutic targets [e.g., RPM1-induced protein kinase (RIPK1), iron] (14, 18, 23–25). Specific molecules require regulatory pathways that involve the Fas-associated protein with a new death domain, caspase-8, caspase-1, RIPK3, mixed lineage kinase domain-like pseudokinase (MLKL), nuclear factor-κB (NF-κB), etc. (26).

β-coronavirus proteins or infection with the complete virus can lead to coronavirus-induced cell death, necroptosis, and pyroptosis (27). Infection with SARS-CoV-2 activates caspase-8 (a master regulator of pyroptosis and necroptosis) and RIPK3 to initiate inflammatory cytokines within lung epithelial cells (28). Necroptosis depends on forming a molecular complex called the necrosome, which incorporates the phosphorylation of RIPK1, RIPK3, and the recruitment of mixed lineage kinase domain-like pseudokinase (MLKL), and pro–caspase-8 (29). Serum levels of RIPK3 have been demonstrated to be upregulated in patients with COVID-19, suggesting the necroptosis-driven response to host defense of SARS-CoV-2 (16). Pyroptosis depends on inflammasomes, caspase-1/11, gasdermin-D (GSDMD), and the release of interleukin-1β (IL-1 β) and IL-18 (30). Ferroptosis triggered by iron overload or glutathione peroxidase 4 (GPX4) inactivation leads to lipid peroxidation and the release of cell contents (31).

Studies on the molecular mechanism of regulated necrosis have improved our understanding of the role regulated necrosis plays in COVID-19. Although the essential functions of regulated necrosis in the immune system are fully described, their roles in COVID-19 are still quite complex and elusive. For example, necroptosis is vital in maintaining T-cell homeostasis (32). Furthermore, excess activated T cells can be removed after clonal expansion, while deregulation can result in immunodeficiency or autoimmunity (32). This review discusses the potential roles and specific consequences of regulated necrosis, including necroptosis, ferroptosis, and pyroptosis, in the pathophysiological process of COVID-19. Furthermore, the mechanism of regulated necrosis and potential therapeutic targets are comprehensively reviewed. The relevance of regulated necrosis in anti-COVID-19 therapy makes it possible to inhibit further infection of COVID-19, in turn giving the development of anti-SARS-CoV-2 drugs a high clinical value.

## 2 Regulated necrosis in COVID-19: A double-edged sword?

Previous studies have shown that regulated necrosis is a double-edged sword in cancer development and progression. It has pro- and antitumor effects that drive oncogenesis and defend against the emergence of cancer, respectively (33). Regarding the SARS-CoV-2 infection process, regulated necrosis results from viral replication in permissive cells, which dominates in the early stage of infection. Therefore, it can inhibit virus invasion by killing cells and activating immune defense function. However, later in the disease, regulated necrosis has extremely pro-inflammatory effects. Pro-inflammatory cytokines (tumor necrosis factor (TNF-α), IFN-γ, IL-1, or IL-6) are the main functional activity of the immune system (18, 20). This process leads to the recruitment of immune cells, the generation of immune complexes, and relevant injury (34). Additionally, the release of processed inflammatory cytokines and the amplification of inflammatory reactions together induce necroptosis and pyroptosis in macrophages, which aggravate lymphopenia through the direct killing of lymphocytes and limit the appropriate immune response (28).

Regulated necrosis can play a protective role in limiting SARS-CoV-2 infection. It seems that SARS-CoV-2 infection manipulates the necrosis process by regulating NF-κ B-dependent cell survival and mitochondria among various pathways to inhibit regulated necrosis and allow SARS-CoV-2 to replicate and spread. Additionally, the anti-SARS-CoV-2 role of regulated necrosis may be mediated by killing cells invaded by SARS-CoV-2, activating innate and adaptive immunity, and promoting apoptosis. In infected cells, the initiation of regulated necrosis, such as necroptosis, ferroptosis, and pyroptosis, rapidly eliminates infected cells to limit the viral spread and avoid harmful host pathogenesis (35). Similarly, regulated necrosis, a third mechanism by which TNF contributes to innate immune control of pathogens, strengthens antiviral responses in regulating virus-induced inflammation and other inflammatory processes (22).

Furthermore, necroptosis releases damage-associated molecular patterns (DAMP) to induce robust cross-priming of CD8^+^ T cells (36). Again, the close interaction between necroptosis and apoptosis also removes infectious cells that terminate virus infection. It seems that apoptosis at the initial stage of the disease wreaks havoc on viral replication (37). Complex IIa induced by deubiquitination of RIPK1 activates caspase-8 and further facilitates apoptosis (13). However, when caspase-8 is blocked, complex IIb is formed and starts necroptosis (13). The two pathways can counteract the upregulation of the cellular FLICE inhibitory protein, an antiapoptotic protein, which defers cell death and benefits SARS-CoV-2 replication (37, 38). Consequently, inhibiting regulated necrosis might be an emerging viral immune evasion strategy.

However, due to the replication and liberation of SARS-CoV-2, regulated necrosis releases a large number of pathogen-associated molecular patterns (PAMP) and damage-associated molecular patterns (DAMP), including inflammatory cytokines and cell contents such as SARS-CoV-2 particles, chemokines, lactate dehydrogenase, adenosine triphosphate (ATP), and reactive oxygen species (ROS). These mediators are beneficial in causing an immune and inflammatory response of the surrounding cells (39, 40). At the same time, adjacent epithelial and endothelial cells are induced to release pro-inflammatory mediators during necrosis (41). In addition, neutrophil infiltration, macrophage activation, Th17, and the high mobility group box-1 protein (HMGB1) promote the inflammatory cascade reaction, which eventually leads to a cytokine storm in host cells and cytokine release syndrome (CRS) in the human body (42–44).

Cytokine storm and inflammatory immune reactions, mainly due to uncontrolled necrosis, occupy a crucial position in COVID-19 complications (45). Due to vascular leakage, inflammatory cells such as T cells, monocytes, and macrophages are recruited from the blood to the lungs (46). Inflammatory mediators such as IL-2, IL-6, and tumor necrosis factor (TNF) are also infiltrated by inflammatory cells into the lungs due to increased production, destroying lung structure (47). In addition, aerobic granular sludge and SARS-CoV-2 RNA spread in the bloodstream, produce immune complexes, and deposit in target organs such as the kidneys, causing a severe inflammatory cascade (48). Furthermore, the cytokine storm reaches other organs through the vascular system (44), leading to multiple organ dysfunction syndromes (MODS) and sequential cell necrosis. For example, pyroptosis and necroptosis are effective mechanisms for the secretion of IL-1β and IL-18, ATP, HMGB1, S100 proteins, and IL-1α, leading to acute phase reactions, inflammatory tissue damage, fever, cytokine release syndrome, neurotoxicity, and potential systemic organ failure in sepsis and other diseases (49).

In addition to regulated necrosis, traditional necrosis can be caused directly by vital pathogenic factors or developed from reversible damage in COVID-19 (13). COVID-19 causes necrotic damage to the lungs, and the resulting cytokine storm can also spread to multiple organs through the circulatory system, resulting in cell necrosis of various organs and leading to MODS (50). Kidney injury and subsequent clinical complications such as hematuria and proteinuria are present in approximately 40% of COVID-19 patients (51). Nephrocortical necrosis is caused by an ACE2 pathway, acute tubular necrosis, and hypercoagulation (51). Neurocyte necrosis is present in patients with acute demyelinating encephalomyelitis (52), and dermatic necrosis in COVID-19 is characterized by infectious exanthemas (41). Data in China showed that one in five patients with COVID-19 has myocardial necrosis (53), with an inflammation response and cytokine storm as possible mechanisms (54). Furthermore, trophoblast necrosis appears in the placentas in pregnant women (55). SARS-CoV-2-induced vasculitis can induce fibrinoid necrosis of small vessel walls (56).

Based on an increasing understanding of the complexity of regulating host cell necrosis in COVID-19 and the connection with the intrinsic immune defense mechanism of SARS-CoV-2 infection (innate immunity of cells), we speculate that regulated necrosis plays a double-edged sword role in COVID-19 and seems to depend on many factors, including downstream effector molecules (various cytokines and chemokines), the specific cell death pathway involved, and the immune response at different clinical stages of COVID-19 progression. However, the driving factors of regulated necrosis still need more studies to fully explain this mechanism.

### 2.1 Necroptosis in COVID-19

Necroptosis is an essential programmed cell death model with a necrotic morphology mediating by RIPK1, RIPK3, and MLKL (57, 58). Previous research has shown that necroptosis could be a double-edged sword during viral infection (59). Necroptosis can lead to cell suicide to prevent viral replication completion and further block disease progression (60). Furthermore, necroptosis stimulates wild antiviral immune responses and accelerates adequate viral clearance from infected organs when apoptosis is inhibited (61). However, with cell rupture, intracellular viruses successfully evade cell death and spread throughout the body (62). Furthermore, uncontrolled lytic cell death can cause tissue injury and severe diseases, including acute respiratory distress syndrome (ARDS), neurodegenerative diseases, and inflammatory diseases (62).

**Figure 1:**
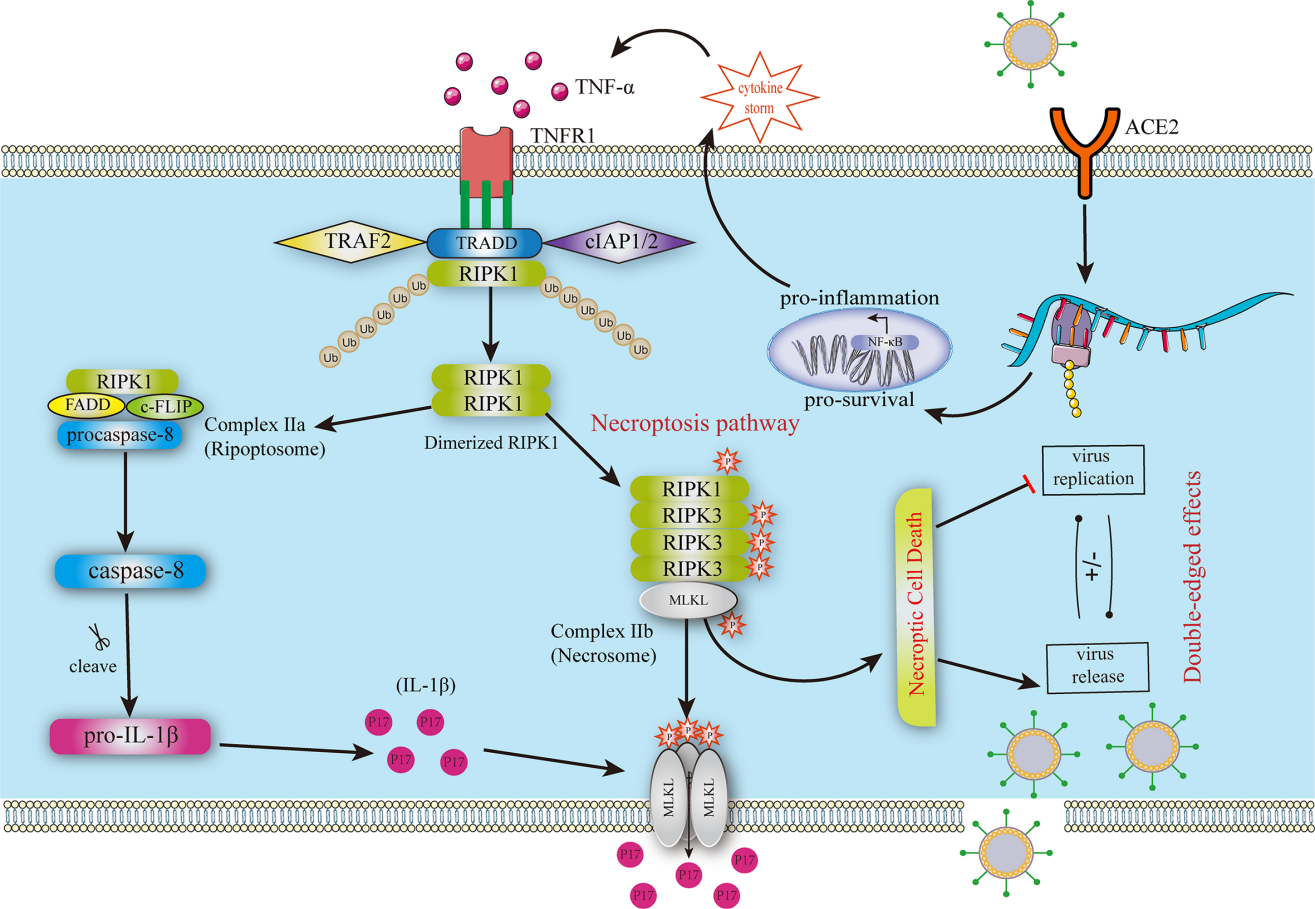
Mechanism of necroptosis in COVID-19. The SRAS-CoV-2 infection leads to severe cytokine storms contributing to necroptosis in uninfected cells. TNF-α binds to TNFR1, forming a stable complex that deubiquitinates and includes complexes II A and II B Complex II b contains phosphorylated RIPK1, RIPK3, and MLKL, triggering the necroptosis pathway. MLKL is oligomerized to form pores in the membrane, leading to cytokine leakage. The other two pathways lead to caspase-8 production, including the RIPK1-independent and the RIPK1-dependent pathways. Then, caspase-8 promotes the production and leakage of IL-1β. FADD, FAS‐associated death domain; MLKL, pseudokinase similar to the mixed lineage kinase domain-like pseudokinase; RIPK1, receptor-interacting protein kinase 1; RIPK3, receptor-interacting protein kinase 3; TNF-α, Tumor Necrosis Factor-alpha; TNFR1, tumor necrosis factor receptor 1; TRADD, TNFR-associated death domain.

Investigating whether SARS-CoV-2 can cause necroptosis begins with the human lung cancer cell line Calu-3 (28). After Calu-3 cells were infected with SARS-CoV-2, MLKL phosphorylation (pMLKL) was up-regulated in infected cells, and antibody staining showed that pMLKL was expressed in the cell membrane. Scientists inactivated infected cells with ultraviolet light, which prevented viral replication and phosphorylation of MLKL, suggesting that necroptosis is virus-dependent (28). Excessive immune response and cytokine production were discovered at the SARS-CoV-2 infection site (10). Necroptosis creates an abundant inflammatory environment by releasing DAMPs to recruit immune cells and chemokines or cytokines to prevent virus infection (63). Critical cytokines discovered in the SARS-CoV-2 condition include TNF-α, IL-1β, and IL-6, all common cytokines released in necroptosis (64–66). However, these robust inflammatory responses lead to complications in COVID-19 patients, such as ARDS, vascular injury, and neurological sequelae leading to physiological deterioration and death (67–69). The discussion elaborates on the double-edged sword effects of necroptosis in COVD-19. The mechanism of necroptosis in COVID-19 is described in **Figure 1**. However, relevant research in this field is still relatively scarce, and detailed mechanisms need to be explored.

As one of the critical cell death pathways in SARS-CoV-2 infection, necroptosis can be orchestrated to alleviate the damage the cytokine storm causes. Therefore, some drugs that can specifically inhibit the necrotic progression of COVID-19 will hopefully contribute to the treatment of COVID-19. Necrostatin-1 (Nec-1) is a specific RIPK1 inhibitor that suppresses the necroptosis signaling pathway. Recently, Nec-1 has been found to prevent COVID-19 complications potentially (23). Furthermore, Nec-1 can alleviate the release of DAMP and pro-inflammatory cytokines, inhibit the inflammatory NF-κB pathway, and reduce ROS damage.

Furthermore, after Nec-1 administration, T cell exhaustion in patients with COVID-19 can be alleviated by modulating host defense (23). However, considering the role of viral promotion in necroptosis, here is the question: Will Nec-1 promote viral infection by inhibiting necroptosis? Relevant studies are needed to explore the connection. Primidone is another inhibitor to block RIPK1 activation and TNF-α-induced necroptosis (70). Necrosulfonamide is an effective inhibitor to decrease pMLKL (59). Additionally, nocodazole, cytochalasin B, and brefeldin A can overtly inhibit pMLKL accumulation in the membrane to prevent rupture of the plasma membrane (71). RIPK3 is a potential target with a predominant increase in SARS-CoV-2 infection (72), but there have been no necroptosis studies relevant for RIPK3 inhibitors. This may be attributed to the fact that RIPK3 also mediates other inflammatory pathways; the removal of RIPK3 does not directly show the performance of necroptosis (73).

### 2.2 Pyroptosis in COVID-19

Pyroptosis, an essential component of the human antiviral innate immune system and one of the critical pathways of programmed cell death after SARS-CoV-2 infection, depends on recognising pattern recognition receptors and the activation and assembly of the inflammasome. Inflammasome activation triggers the influx of sodium ions and water-mediated by gasdermin D, resulting in pyroptosis and the maturation and release of cytokines. PAMPs and DAMPs, such as SARS-CoV-2 RNA, activate cytoplasmic pattern recognition receptors (74), among which the NACHT, LRR, and PYD domains contain protein 3 (NLRP3), an essential receptor that causes pyroptosis.

Pyroptosis may play a dual role in antiviral and viral promotion in the COVID-19 process: pyroptosis is critical for the induction of effective antiviral immune responses and disease resolution to limit pathogen infection and kill viruses-invading cells. A protective role of inflammasome signaling and IL-1β release has been demonstrated against multiple pathogens, particularly in the acute phase of the disease (75). Studies in patients with mild asymptomatic COVID-19 suggest that the activation of inflammasomes and pyroptosis may benefit the host due to the initiation of the protective response against SARS-CoV-2. However, its prolonged late-stage activation and uncontrolled pyroptosis may be the basis for immunopathology with the rapid release of a substantial number of PAMPs (SARS-CoV-2 particles, cell contents, etc.), DAMPs (ATP, ROS, etc.), and excessive production of pro-inflammatory cytokines (IL-1α/β, IL-6, TNF, etc.) and chemokines into the inflammatory microenvironment to recruit more immune cells (39). The activation of macrophages and HMGB1, as well as neutrophil infiltration, can promote the inflammatory chain reaction of surrounding cells, causing an excessive immune response and tissue damage that eventually leads to severe COVID-19 and an increased risk of ARDS (39, 42–44, 75). ARDS results from dysregulated hyperinflammation, rather than viral replication or infection that causes lung injury (76).

**Figure 2:**
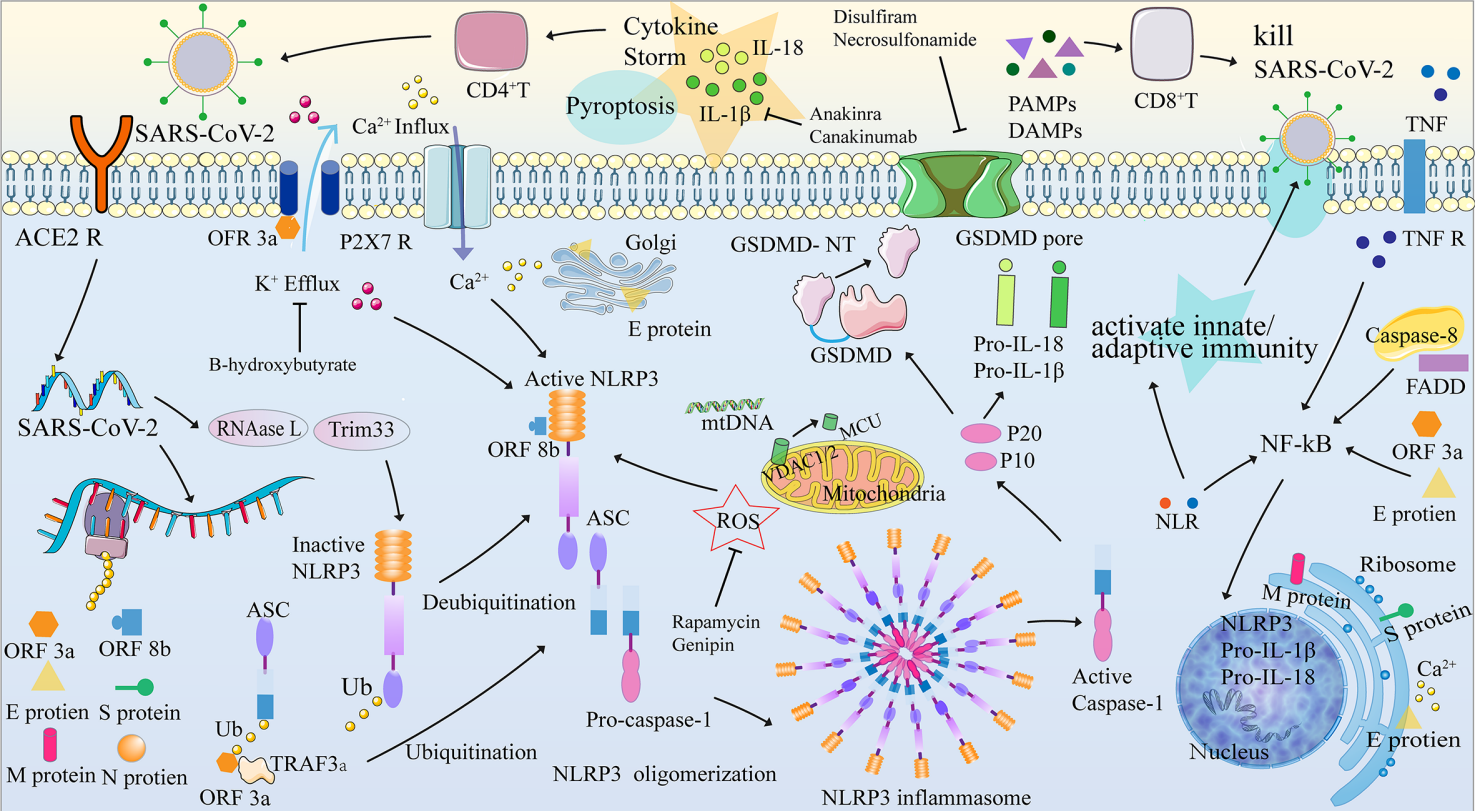
NLRP3 inflammasome, pyroptosis, and their mechanisms in COVID-19. The pyroptosis process can be divided into four stages: (1) activation, (2) formation of the inflammasome, (3) release of IL-1 β and IL-18, and (4) GSDMD-induced pyroptosis. When the SARS-COV-2 protein binds to the ACE2 receptor and is internalized by endocytosis, the SARS-COV-2 RNA is translated and replicated, transcribing the structural proteins ORF3a, ORF8b, and SARS-COV-2 (N, S, M and E proteins). The NLRP3 inflammasome assembly signal, provided by PAMP and DAMP, is involved in the imbalance of intracellular ion concentration (Ca^2+^ concentration increased and K^+^ concentration decreased), mitochondrial dysfunction leading to release of ROS, Ca^2+^ and mtDNA, cardiolipin translocation of cardiolipin from inner to the outer mitochondrial membrane, phagocyte and lysosome rupture that releases cathepsin, etc. Activation of NLRP3 results in recruitment, oligomerization, and binding to ASCs, which then recruit and bind to pro-caspase-1 through their shared domain to drive the assembly of NLRP3-ASC-procaspase-1. The formation of the NLRP3 inflammasome leads to two results: (1) the release of IL-1 β and IL-18 and (2) GSDMD-induced pyroptosis. ACE2R, Angiotensin converting enzyme 2 receptor; ASC, Apoptosis-associated speck-like protein containing a caspase recruitment domain (ASC); ATP, adenosine triphosphate; BTK, Bruton’s tyrosine kinase; Ca/CaMK, Calcium/Calmodulin-dependent protein kinase; CpG, Cytosine Phosphate Guanosine; DAMPs, damage-associated molecular patterns; ER, endoplasmic reticulum; FADD, Fas-associated death domain; Golgi, Golgi apparatus; GSDMD-NT, gasdermin D-N terminal; IL, Interleukin; IRAK, Interleukin-1 receptor-associated kinase; LRR, leucine-rich repeat; MCU, mitochondrial Ca^2+^ uniporter; MtDNA, mitochondrial DNA; MyD88, myeloid differentiation factor 88; NF-κB, Nuclear factor-κB; NLRP3, NLR family, pyrin domain containing 3; NOD, nucleotide-binding and oligomerization; ORF3a, Open reading frame 3a; ORF8b, Open reading frame 8b; PAMPs pathogen-associated molecular patterns; PRR, pattern recognition receptor; P2X7R, purinergic ligand-gated ion channel 7 receptor; RNAase L, latent endoribonuclease; ROS, reactive oxygen species; TNF, tumor necrosis factor; TNFR, tumor necrosis factor receptor; TRAF3, TNF receptor-associated factor 3; Trim33, Tripartite motif-containing protein 33; TLR 4, Toll-like receptor 4; JAKUb, Ubiquitin; VDAC1/2, voltage-dependent anion-selective channel ½.

Additionally, SARS-CoV-2 antigens and RNA are disseminated in the bloodstream after pyroptosis and likely produce immune complexes that deposit in target organs, such as the kidneys, to induce a severe inflammatory cascade (48) and may explain the poor prognosis of severe COVID-19. Besides, many biomarkers in the models of death in COVID-19 have been described in **Table 1** to predict immune responses and further evaluate the prognosis. Pyroptosis is also the key cause of overexuberant inflammation and cytokine release observed in severe and fatal cases of COVID-19, damage to the lung endothelium accompanied by immune cell infiltration, and systemic hypercoagulability (88). Targeting the NLRP3 Inflammasome and mitigating aberrant inflammatory responses may play an important role in severe COVID-19 and complications of SARS-CoV-2 infection (48). Mechanisms, potential targets, and double-edged sword effects of the NLRP3 inflammasome and pyroptosis in COVID-19 are described in **Figure 2**.

**Table 1:** Biomarkers in the modes of cell death in COVID-19.

Cell death modes	Biomarkers	Functions	References
**Necroptosis**	RIPK3	The serum RIPK3 level is high in COVID-19 patients in severe cases but does not directly indicate necroptosis.	(72)
	MLKL	Robust phosphorylation of MLKL shows that necroptosis occurs.	(77)
	IL-1β	Demonstrates the development and severity of COVID-19.	(28, 78)
	RIPK1	Elevated RIPK1 levels show apoptosis or necroptosis, or both.	(70)
**Ferroptosis**	F4-hydroxynonenal (4-HNE) Cytotoxic malondialdehyde	Product of ferroptosis lipid peroxide degradation. Cytotoxic and damage heart cells, resulting in cardiac dysfunction and heart failure.	(79, 80)
	Serum ferritin	Increase related to severity and mortality risk in COVID-19 patients.	(6, 81)
	Transferrin receptor (TfR)	TfR expression plays a bridge role between iron overload and the gender and age difference in the severity of COVID-19.	(82)
	Total iron, nonheme iron in the lavage fluid, and intracellular Tf and lactoferrin (Lf)	Increase in Acute Respiratory Distress Syndrome (ARDS) patients.	(83)
**Pyroptosis**	IL-6	Significant increase in non-survivors vs. survivors and severe vs. non-severe disease; connected to cytokine storm, additional tissue damage, and multiple organ failure (MOF).	(84)
	ASC	Forms the NLRP3 inflammasome.	(85)
	IL-1β	The most severe patients are correlated with increased pro-inflammatory cytokines, evocative of a cytokine storm.	(85)
	IL-18/IFN-γ	Activates macrophages, resulting in multiple cytokine release, hemophagocytosis, coagulopathy, and ARDS.	(86)
	miRNAs (miR-223-3p)	Regulates the NLRP3 inflammasome and acts at a priming and activation level of NLRP3 formation.	(87)

ASC, Apoptosis-associated speck-like protein containing a caspase recruitment domain; COVID-19, Coronavirus Disease 2019; IFN, interferon; IL, interleukin; MLKL, mixed lineage kinase domain-like pseudokinase; NLRP3, NLR family, pyrin domain containing 3; RIPK1, receptor-interacting protein kinase 1; RIPK3, receptor-interacting protein kinase 3.

A deep understanding of the molecular mechanisms and role of pyroptosis will allow selective interference of the deleterious actions of pyroptosis in pathological contexts and promote the beneficial effects for therapeutic purposes. Many potential therapeutic agents and their targets have been studied to manipulate the COVID-19-related immune response and pyroptosis, summarized in **Table 2**. As the mechanisms of pro-infective activity by pyroptosis are well understood, inhibiting the pyroptosis process can limit intracellular replication of SARS-CoV-2 and inhibit extensive cytokine storm and tissue inflammation induced by the NLRP inflammasome by causing pyroptosis, which is expected to be used in COVID-19 treatment (88). Disulfiram and necrosulfonamide, effective inhibitors of pyroptosis and GSDMD pore formation (88, 103), can limit intracellular virus replication and inhibit extensive cytokine storm and tissue inflammation by causing apoptosis, which is also expected to be used in COVID-19 treatment (104). A phase 2 randomized (2:1), double-blind placebo-controlled trial of disulfiram evaluates the effect of disulfiram on the severity of COVID-19 symptoms, the viral load of SARS-CoV-2, and biomarkers of inflammation and pyroptosis over 31 days. (ClinicalTrials.gov Identifier: NCT04485130). Furthermore, rapamycin and genipin can significantly inhibit the expression level of the NLRP3 inflammasome-related protein (93) or alleviate the inflammatory response by activating the antioxidant system (24). Recently, a single-center double-blind placebo-controlled randomized clinical trial assessed the clinical effectiveness of rapamycin in minimizing or decreasing the severity of (acute lung injury/ARDS) in participants infected with mild to moderate COVID-19 (ClinicalTrials.gov Identifier: NCT04482712). B-hydroxybutyrate can inhibit K^+^ outflow and caspase-1 activation (92), which may greatly benefit patients with COVID-19. A randomized placebo-controlled double-blind cross-over acute intervention study showed that beta-hydroxybutyrate had acute beneficial hemodynamic effects in patients with heart failure and healthy controls (ClinicalTrials.gov Identifier: NCT04573764).

**Table 2:** Treatment targets and mechanisms in the modes of cell death in COVID-19.

Cell death mode	Potential therapeutic targets	Treatment pathways and mechanisms	Potential drugs	References
**Necroptosis**	RIPK1	Inhibits RIPK1 specifically	Nec-1	(23)
		Blocks RIPK1 activation and further blocks the TNFα-induced necroptosis	Primidone	(70)
	MLKL	Decreases the phosphorylation of MLKL	Necrosulfonamide (NSA)	(59)
		Inhibits pMLKL accumulation in the membrane to prevent the plasma membrane from disintegration	Nocodazole, Cytochalasin B, and Brefeldin A(NCB)	71)
	TNF-α and IFN-γ	Blocks TNF-α and IFN-γ to alleviate necroptosis in COVID-19	Neutralizing antibody (Anti-TNF-α, Anti-IFN-γ)	(77)
	Anexelekto (AXL)	Oppresses the p38/mitogen-activated protein kinase (MAPK) pathway and further reduces cytokine production and virus replication	Gilteritinib	(89)
	NF-κB pathway	Inhibits NF-κB pathway and reduces ROS damage.	Nec-1	(23)
	DAMPs and pro-inflammatory cytokines	Alleviates the release of DAMP and pro-inflammatory cytokines.	Nec-1	(23)
**Pyroptosis**	Release of cytokine	Inhibits the cytokine storm. Improves the survival rate of COVID-19.	IL-1 inhibitor (Anakinra) half-life-prolonged IL-1β (canakinumab) rilonacept	(25, 90, 91)
	ROS	Alleviates inflammatory reaction by activating the antioxidant system.	Rapamycin, genipin, agrabine, and resveratrol	(24)
	ASC oligomerization	Blocks ASC oligomerization. Inhibits K^+^ efflux and caspase-1 activation.	B-hydroxybutyrate (BHB)	(92)
	NLRP3 oligomerization	Binds to the NACHT domain of NLRP3 to inhibit its oligomerization.	Tranilast	(24)
		Inhibits the expression level of NLRP3 inflammasome-related proteins.	Rapamycin, genipin, agrabine, and resveratrol	(93)
	Autophagy	Induces autophagy. Inhibits macrophage mitochondrial damage.	Resveratrol, HU-433 and HU-308.	(94, 95)
**Ferroptosis**	System Xc-	Inhibits system Xc- to cause intracellular glutathione (GSH) depletion, inducing ferroptosis.	Erastian, Sulfasalazine (SAS), Sorafenib, extracellular glutamate accumulation.	(96)
	ROS regulation	Inhibits lipid oxidation and decreases ROS of intracellular lipids.	Lipid antioxidants (vitamin E, Fer-1, and Lip-1).	(97, 98)
		Prevents the formation and scavenging of ROS.	Reducing agents (methemoglobin reductase, ascorbic acid, and glutathione).	(99)
	Iron	Binds to free iron to inhibit its redox properties. Prevents membrane lipid oxidation and the Fenton reaction. Removes iron from iron-binding proteins.	Iron chelators (desferrioxamine, deferoxamine mesylate, and deferrione)	(25, 100, 101)
	Iron autophagy	Inhibits fermodulin.	Analogues of fermodulin-1 and liproxistatin-1.	(102)

ASC, Apoptosis-associated speck-like protein containing a caspase recruitment domain; COVID-19, Coronavirus Disease 2019; DAMP, damage-associated molecular patterns; IFN-γ, interferon-γ; IL, interleukin; MLKL, mixed lineage kinase domain-like pseudokinase; NF-κB, Nuclear factor-κB; Nec-1, Necrostatin-1; NLRP3, NLR family, pyrin domain containing 3; RIPK1, receptor-interacting protein kinase 1; ROS, reactive oxygen species; TNF-α, Tumor Necrosis Factor-alpha.

Although the appropriate phage for patients with COVID-19 to accept pharmacological interventions is worth exploring, much remains obscure regarding potential agonists to provide a host for pyroptosis Downstream cytokine antagonists may have advantages in treating COVID-19, given that the overexpression of IL-1β, specifically in the lungs, is sufficient to recapitulate many of the ARDS phenotypes (90). Anakinra, an inhibitor of IL-1, and canakinumab, a half-life-prolonged IL-1β, may have potential benefits in the treatment of COVID-19, for which prospective randomized controlled trials are currently underway (90). RNA sequencing of the early recovery stage of COVID-19 showed that classical CD14^++^ and CD14^++^ IL-1β^+;^monocytes are of greater abundance with high expression of inflammatory genes, indicating that IL-1β may be a potential target for the COVID-19 intervention (105). Furthermore, existing retrospective cohort trials have shown that high doses of anakinra, with or without dexamethasone, can improve the survival rate of COVID-19 (25, 91). However, a prospective study did not show clinical results and required further investigation (106).

### 2.3 Ferroptosis in COVID-19

**Figure 3:**
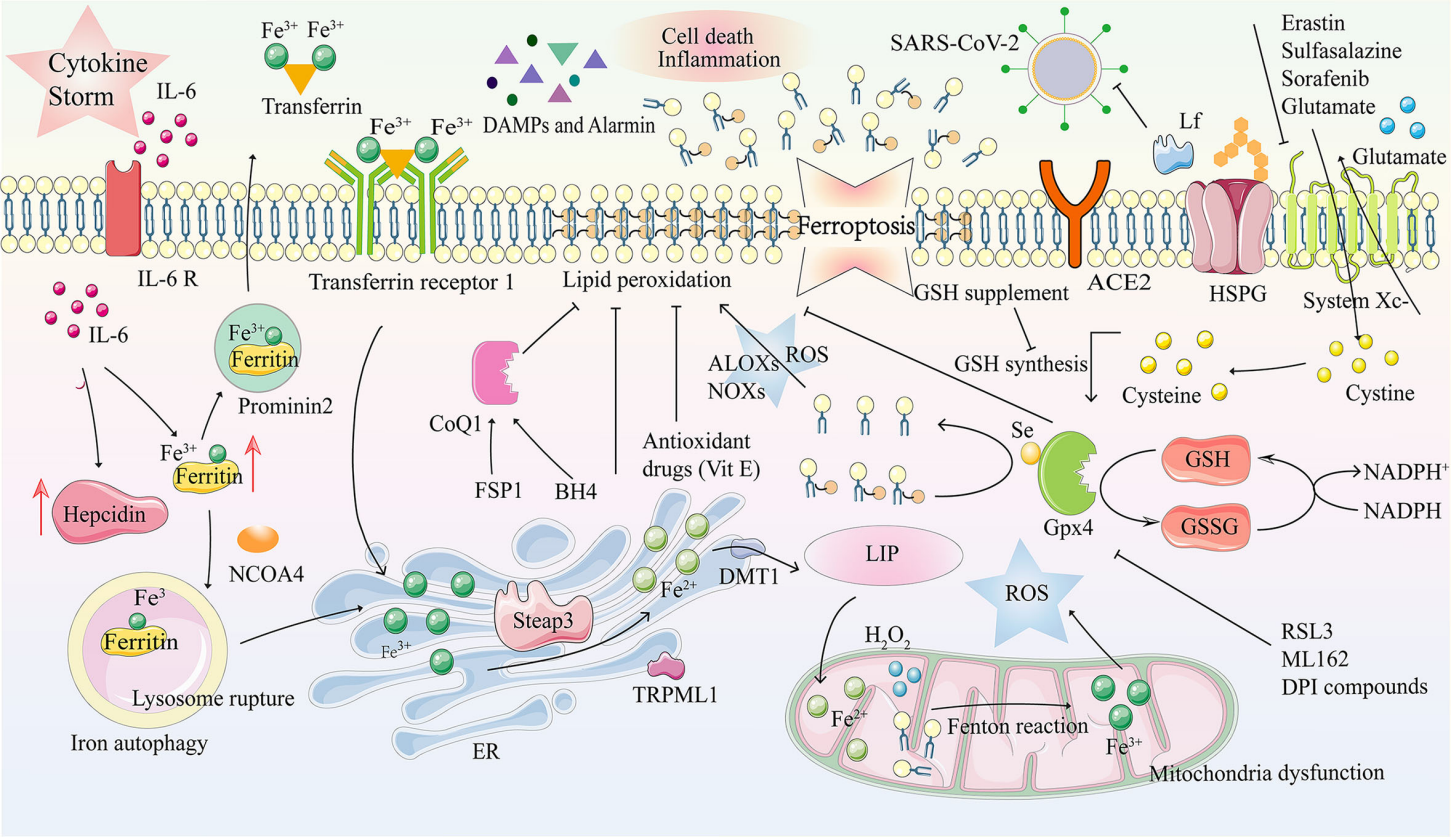
Ferroptosis and its mechanism in COVID-19. Ferroptosis is a kind of programmed cell death characterized by an imbalance in intracellular iron metabolism or a distortion of the glutathione peroxidation pathway. The transferrin receptor recognizes excess transferrin carrying Fe^3+^ and enters cells through endocytosis after SARS-COV-2 infection. Metal reductase Steap3 reduces Fe ions from trivalent to divalent, while iron channels DMT1 and TRPML1 in the endosome membrane transport Fe^2+^ to the cytoplasm, accompanied by iron accumulation. In the case of intracellular iron overload, chemical substances in the mitochondrial electron transfer chain react with H2O2, Fe ^2+^, and lipids, together inducing the Fenton reaction, which produces large amounts of ROS. Due to GPX4 depletion and iron overload in LIP, lipid, nucleic acid, and protein peroxidation results in cell membrane damage due to oxidative stress and ferroptosis. DAMPs and Alarmin (HMGB1, IL-33, TNF) are released, eventually aggravating cell death and inflammation. Tf and prominin 2 can effectively excrete iron from cells and inhibit ferroptosis. ACE2R, Angiotensin-converting enzyme 2 receptor; ALOXs, arachidonate lipoxygenases; BH4, tetrahydrobiopterin; CoQ10, coenzyme Q10; DAMP, damage-associated molecular patterns; DFO, deferoxamine; DMT1, divalent metal transporter 1; DPI, diphenyleneiodonium; ER, endoplasmic reticulum; FSP1, ferroptosis suppressor protein 1; GPX4, glutathione peroxidase 4; GSH, glutathione; GSSG, oxidized GSH; HMGB1, high mobility group box-1 protein; HSPGs, heparan sulfate proteoglycans; H2O2, hydrogen peroxide; IL, interleukin; Lf, lactoferrin; LIP, labile iron pool; LOXs, lysyl oxidases; NADP^+^, nicotinamide adenine dinucleotide phosphate; NADPH, nicotinamide adenine dinucleotide phosphate; NCOA4, nuclear receptor coactivator 4; NOXs, NADPH oxidases; PLOOH, phospholipid hydroperoxide; PUFAs, polyunsaturated fatty acids; ROS, reactive oxygen species; RSL3, 1S,3R-RSL3; Se, selenocysteine; Steap3, six-transmembrane epithelial antigen of prostate 3; TFR1, transferrin receptors 1; TNF, tumor necrosis factor; TRPML1, transient receptor potential mucolipin 1; VitE, vitamin E.

Due to intracellular iron overload caused by many factors such as hepcidin, transferrin receptor (TfR), free iron release, and inhibition of GPX4 (107–111), ferroptosis is an iron-dependent programmed cell death separate from pyroptosis. It is characterized by an increase in membrane lipid peroxide levels and is accompanied by a decrease in glutathione and GPX4 expression levels (112–115). Therefore, the imbalance of intracellular iron homeostasis caused by SARS-CoV-2, which leads to fatal ferroptosis, is closely related to the mechanism of COVID-19 and the pathophysiological process of various complications. The means of ferroptosis in COVID-19 are described in **Figure 3**.

More and more evidence has shown that excessive inflammatory reactions and complications caused by COVID-19, such as acute respiratory distress, heart and kidney injury, and dysfunction of the blood and immune system, are closely related to oxidative stress and ferroptosis (116, 117). Elevated serum ferritin levels caused by COVID-19-related hyperinflammation are likely to trigger further tissue damage (116). Ferroptosis of immune cells during infection is considered immunogenic and pro-inflammatory, beneficial for disease progression, and causes cell death, which may explain the clinical characteristics of MODS in COVID-19 (118). Cell death can induce the release of DAMPs, PAMPs, and alarmins recognized by immune receptors and, finally, aggravate ferroptosis and inflammation (118). One of the main processes in the pathogenesis of COVID-19 infected with SARS-CoV-2 may be the imbalance of intracellular iron homeostasis and fatal ferroptosis (119). Four possible factors mainly induce this process: massive release of the iron homeostasis regulator Hepcidin, excessive iron influx dependent on the transferrin receptor (TfR), which mediates cellular iron uptake through endocytosis of iron-loaded transferrin during SARS-CoV-2 replication, attack of hemoglobin to release free iron into the circulation, and inhibition of GPX4 by SARS-CoV-2 (107–109).

ROS, reactive nitrogen species, and reactive sulfur species (116, 120, 121) produced during pathological conditions can cause oxidative damage and ischemia-reperfusion injury to different organs, such as the lungs, liver, kidney, heart, intestine, and brain (122–124). To a great extent, ferroptosis is associated with hyperinsulinemia, hypercoagulable state, hemorrhagic stroke injury, shock, and MODS (123, 125, 126). Iron overload is also cardiotoxic; the Haber-Weiss and Fenton reactions produce harmful hydroxyl radicals that increase ROS levels in the heart and oxidative stress. Malondialdehyde and F4-hydroxynonenal are cytotoxic and damage heart cells, leading to cardiac dysfunction and heart failure. Furthermore, iron overload may play a role in the hypercoagulable state of patients with severe COVID-19. The destruction of hemoglobin increases the amount of free iron in the blood, leading to iron-induced oxidative stress, thrombocytosis, and changes in red blood cell viscosity. Then fibrinogen is transformed into fibrin clots, leading to pathological thrombosis. Furthermore, hemoglobin loses its ability to bind to oxygen and deliver to major organs, leading to multiple organ failures (127).

Previous studies have shown that iron overload plays a vital role in the pathogenesis of multiple system diseases caused by SARS-CoV-2 infections (31). Reducing iron levels in infected cells can effectively inhibit virus growth and disease progression caused by SARS-CoV-2 (128). Lipophilic antioxidants, including vitamin E, ferrostatin-1, and liproxstatin-1, can alleviate ferroptosis by inhibiting lipid autoxidation, which may have potential value in treating COVID-19 (97, 98). Iron chelators, such as deferoxamine, deferoxamine mesylate, and deferiprone, can bind to free iron to inhibit its redox properties, prevent the Fenton reaction, down-regulate hepcidin, remove iron from iron-binding proteins, etc. (100, 101, 128). Treatment of COVID-19 with reducing agents, including methemoglobin reductase, ascorbic acid, and glutathione to prevent formation and scavenging, may be of great value (99). A new generation of ferroptosis inhibitors, such as improved ferrostatin-1 and liproxstatin-1 analogs, may also be potential drug candidates for COVID-19 (102). The process and therapeutic targets of ferroptosis in COVID-19 are described in **Figure 3**.

## 3 Conclusions

In conclusion, this review elaborates on the biochemical and molecular mechanisms of regulated necrosis in COVID-19 and introduces the latest research progress. Furthermore, we discuss the two sides of regulated necrosis to provide a new view on the treatment of COVID-19. After analyzing the difference between traditional and regulated necrosis, we showed the overall effects of necrosis on the pathophysiology of COVID-19. However, to better understand the two sides of regulated necrosis, future research should investigate the following problems: (i) the driving factors of activation in regulated necrosis in COVID-19; (ii) the possible mechanism of how inflammatory molecular interaction and the influence of the inflammatory environment caused by SARS-CoV-2 affect the regulation of regulated necrosis; and (iii) the effects of regulated necrosis on the regulation of immune cells, antiviral immunity, and the efficiency of targeted therapy. In clinical use, diagnostic criteria, disease severity classification system, combined antiviral treatment, secondary infection, and cytokine measurement should be considered. The disease severity classification system is vital to prompt the initiation of immunomodulatory therapy, which may be beneficial only for severe cases of COVID-19. Still, aggressive anti-inflammatory therapy can prevent progression in mild or moderate patients. The timing of treatment, the variability in the course of the disease, and the proper patient stratification will be essential for identifying the ‘sweet spot’ (appropriate time and stage of inflammasome-inhibiting interventions). In short, understanding the double-edged sword of regulated necrosis with SARS-CoV-2-manipulated molecular details and applying drugs to target regulated necrosis or its downstream pathway alone or in combination with other immunotherapy methods will have great potential as a new treatment method for COVD-19. With further studies in progress, promising results will emerge in the future.

## Author contributions

CS, YH, and RZ: writing the initial draft preparation, table and figure production in equal contribution, with the help of JinW, YZ, and XC; JMW and JiaW: conceptualization, review, and critical revision. All authors: literature search, review, commentary, and final approval of the manuscript.

## Funding

JMW was supported by the Zhengzhou University Education and Teaching Reform Research and Practice Project (2021ZZUJGLX219). This research was also supported in part by the Intramural Research Program of the National Human Genome Research Institute, National Institutes of Health.

## Conflict of interest

The authors declare that the research was conducted in the absence of any commercial or financial relationships that could be construed as a potential conflict of interest.

## Publisher’s note

All claims expressed in this article are solely those of the authors and do not necessarily represent those of their affiliated organizations, or those of the publisher, the editors and the reviewers. Any product that may be evaluated in this article, or claim that may be made by its manufacturer, is not guaranteed or endorsed by the publisher.
